# MicroRNA‐210 level as a biomarker for pre‐eclampsia severity: A systematic review and meta‐analysis

**DOI:** 10.1002/ijgo.71145

**Published:** 2026-06-23

**Authors:** Dávid Márai, Isabel Pinto Amorim das Virgens, Ádám Vincze, Mahmoud Obeidat, Péter Hegyi, Mikolt Bakony, Zsófia Réka Erdélyi, Zsolt Melczer, Sándor Valent

**Affiliations:** ^1^ Center for Translational Medicine Semmelweis University Budapest Hungary; ^2^ Department of Obstetrics and Gynecology Semmelweis University Budapest Hungary; ^3^ Institute for Translational Medicine, Medical School University of Pécs Pécs Hungary; ^4^ Institute of Pancreatic Diseases Semmelweis University Budapest Hungary

**Keywords:** microRNA, miR‐210, miRNA, miRNA‐210, PE, pre‐eclampsia, prognostic marker, severe PE

## Abstract

**Background:**

Pre‐eclampsia is a serious pregnancy‐related syndrome lacking accurate prognostic markers. MicroRNA‐210, overexpressed in the circulation of women with pre‐eclampsia, has recently emerged as a promising biomarker.

**Objectives:**

We conducted a systematic review and meta‐analysis to evaluate the relationship between miR‐210 levels and pre‐eclampsia at different degrees of severity.

**Search Strategy:**

The study was prospectively registered on PROSPERO (CRD42024604698) and included a comprehensive systematic search of five databases conducted on November 21, 2024.

**Selection Criteria, Data Collection and Analysis:**

The included studies involved pregnant women with mild or severe pre‐eclampsia compared to normotensive controls, meeting guideline‐based diagnostic criteria and reporting circulating microRNA‐210. Precalculated effect sizes were pooled to obtain a weighted mean and 95% confidence interval (CI) for microRNA‐210‐fold increase. Subgroup analysis assessed pre‐eclampsia severity, using an inverse variance method in random‐effects meta‐analysis models.

**Main Results:**

Our search identified 776 records, of which seven were deemed eligible for analysis. The fold change values, reflecting the increase in microRNA‐210 levels between the control and pre‐eclamptic populations, were 3.33 (CI: 0.69–5.98; *I* ^2^ = 93%, CI: 88–96%) in the mild pre‐eclampsia group and 7.23 (CI: 2.27–12.18; *I* ^2^ = 98%, CI: 98–99%) in the severe pre‐eclampsia group, indicating a significant effect (*P* = 0.01) in the subgroup difference tests. The mean ratio of microRNA‐210‐fold change between the two severity groups was 2.05 (CI: 1.41–2.69; *I* ^2^ = 85%, *P* < 0.01), favoring the severe population.

**Conclusion:**

Elevated microRNA‐210 levels are strongly associated with pre‐eclampsia severity at the time of diagnosis, with a consistent, significant stepwise increase from mild to severe disease. Whether miR‐210 holds predictive value earlier in pregnancy remains to be established in prospective studies with first‐trimester sampling.

**Abbreviations**
ACOG: American Congress of Obstetricians and Gynecologists
CI: confidence interval
ddPCR: droplet digital polymerase chain reaction
DNA: deoxyribonucleic acid
FC: fold change
FGR: fetal growth restriction
ISSHP: International Society for the Study of Hypertension in Pregnancy
IUGR: intrauterine growth restriction
JBI: Joanna Briggs Institute
miR‐210: microRNA‐210, Hsa‐miR‐210‐3p
miRNAs: microRNAs
MOOSE: meta‐analysis of observational studies in epidemiology
PE: pre‐eclampsia
PRISMA: Preferred Reporting Items for Systematic Reviews and Meta‐Analyses
qRT‐PCR: quantitative reverse transcription polymerase chain reaction
RNA: ribonucleic acid
sFLT: soluble fms‐like tyrosine kinase‐1
sFlt‐1/PlGF ratio: soluble fms‐like tyrosine kinase‐1/placental growth factor

## 1 INTRODUCTION

### 1.1 Background

Pre‐eclampsia (PE) is a pregnancy‐related syndrome that affects approximately 4.6% of pregnancies worldwide and causes approximately 500 000 fetal and 70 000 maternal deaths per year.^1^, ^2^, ^3^, ^4^ The exact definition of the disease is not universally accepted.^5^ According to the International Society for the Study of Hypertension in Pregnancy (ISSHP), PE is diagnosed by the presence of de novo hypertension after 20 weeks of gestation, accompanied by proteinuria and/or evidence of maternal end‐organ or uteroplacental dysfunction.^2^ Traditionally, PE has been categorized based on onset timing as either early (<34th week of gestation) or late (>34th week of gestation), and classified as mild or severe according to clinical severity.^6^ The American Congress of Obstetricians and Gynecologists (ACOG) classifies mild PE as blood pressure >140/90 mmHg with end‐organ damage without any severe features, whereas severe PE is diagnosed with blood pressure >160/110 mmHg and/or signs of significant end‐organ dysfunction.^7^

Severe PE increases the risk of life‐threatening complications, preterm delivery, intrauterine growth restriction (IUGR)/fetal growth restriction (FGR), and maternal mortality.^8^ PE can progress rapidly without warning, and blood pressure levels alone are not a reliable indicator of stratifying immediate risk.^2^ Criteria for hospitalization are not well defined, and the prognosis of the disease is challenging to predict.^2^, ^7^

Despite advances in understanding the molecular basis of PE, much about its pathogenesis remains unclear, with multiple theories attempting to explain it.^9^ The two‐stage model attributes placental dysfunction to syncytiotrophoblast stress, leading to oxidative stress, mitochondrial damage, release of proinflammatory cytokines and antiangiogenic factors such as soluble fms‐like tyrosine kinase‐1 (sFLT).^9^, ^10^ Consistent with this model, pre‐eclamptic placentas also exhibit hypoxia‐associated metabolic dysfunction, further supporting the role of impaired placental adaptation to reduced oxygen availability in PE pathogenesis.^11^ Elevated sFLT binds to vascular endothelial growth factor (VEGF) more effectively, decreasing its bioavailability and nitric oxide (NO) production, culminating in vasoconstriction and endothelial dysfunction.^9^, ^10^, ^12^ Insufficient NO production is associated with PE, with the placental endothelial nitric oxide synthase (eNOS) isoform playing a pivotal role.^13^

Oxidative stress during PE activates hypoxia‐driven gene expression, leading to the release of exosomes containing various micro‐ribonucleic acids (miRNAs).^12^ MiRNAs are non‐coding, single‐stranded RNAs ranging from 19 to 25 nucleotides.^14^ MiR‐210 (homo sapiens micro‐ribonucleic acid‐210‐3p (Hsa‐miR‐210‐3p)) is the most frequently associated miRNA with PE, overexpressed in placental tissue and circulation, and has been identified as a central regulatory node in its pathophysiology.^15^, ^16^ Studies indicate that miR‐210, upregulated under hypoxic conditions, primarily in a hypoxia‐inducible factors (HIF)‐dependent manner, inhibits cell proliferation, mitochondrial respiration, DNA (deoxyribonucleic acid) repair, and angiogenesis.^17^

Circulating miR particles, notably miR‐210, demonstrate stability and have recently been recognized as promising biomarkers for PE.^12^, ^18^ There is a need for an effective prognostic and predictive marker for PE in both early and late pregnancies. Although a number of potential markers have been explored as optimal biomarkers, currently no single test can reliably identify individuals at risk of developing PE by term with the necessary performance for widespread use.^19^

### 1.2 Objectives

Given the central role of miR‐210 in the pathophysiology of PE and its consistent overexpression in affected pregnancies, we conducted a systematic review and meta‐analysis to evaluate the association between circulating miR‐210 levels and the severity of PE, aiming to identify it as a potential biomarker for the condition.

## 2 METHODS

Our systematic review and meta‐analysis were conducted in accordance with the Preferred Reporting Items for Systematic Reviews and Meta‐Analyses (PRISMA) 2020 statement^20^ (Table S2), the Meta‐analysis Of Observational Studies in Epidemiology (MOOSE) guidelines^21^ Supporting Information (Method 1.4), and the methodological guidance outlined in the Cochrane Handbook for Systematic Reviews of Interventions.^22^ The study protocol was registered on PROSPERO^23^ (CRD42024604698), and was fully adhered to.

### 2.1 Eligibility criteria

We used the Population, Exposure, Comparator, and Outcome (PECO) framework to define eligibility criteria.^24^ The population comprised pregnant women, and the exposure of interest was PE of varying severity. Eligible studies were required to report a diagnostic definition for PE, preferably according to established criteria such as those of the ACOG or ISSHP, and to include mild and/or severe PE subgroups. The comparator group consisted of healthy, normotensive pregnant women without pregnancy complications. The outcome of interest was the difference in circulating miR‐210 levels, measured in plasma or serum, compared with those in the control population. Case–control and cohort observational studies, including prospective and retrospective designs, were eligible for inclusion. Studies were excluded if they assessed only postpartum placental tissue rather than circulating plasma or serum miR‐210, because the aim of this review was to evaluate a potentially clinically accessible biomarker. We also excluded studies that did not provide a clear diagnostic definition of PE, did not distinguish PE severity groups, or did not include mild and/or severe PE subgroups, lacked a healthy normotensive control group, or did not report extractable quantitative data with measures of variability required for meta‐analysis. Studies focusing exclusively on other hypertensive disorders of pregnancy or on pregnancy complications unrelated to PE were excluded. Case reports, case series, conference abstracts, gray literature, and reviews were also excluded. Non‐English articles were translated and assessed for eligibility.

### 2.2 Information sources

We performed a systematic search of five databases: MEDLINE (via PubMed), Embase, Cochrane Central Register of Controlled Trials (CENTRAL), Scopus, and Web of Science, up to November 21, 2024. No filters or restrictions were applied during the search. Additionally, backward and forward citation searching was performed utilizing a reference‐checking tool to identify all relevant references meeting our eligibility criteria.^25^

### 2.3 Search strategy

The primary search strategy used during the systematic search focused on the following domains: population, exposure, and outcome. The complete search key is provided in the Supporting Information (Method 1.3 Search Key).

### 2.4 Study selection

Two independent review authors (D.M. and A.V.) conducted the selection process at each stage using the reference management program EndNote 20^26^ and the Rayyan application.^27^ The stages included duplicate removal, title‐abstract selection, and full‐text selection. Any disagreements were resolved by a third author (Zs.E.). Cohen's kappa coefficient (κ)^28^ was used to assess the level of agreement.

### 2.5 Data extraction

Data were independently collected from eligible articles by two authors (D.M. and Zs.E.) using a predefined Microsoft Excel spreadsheet (Windows 10). A third author (A.V.) resolved conflicts in case of disagreement. Whenever possible, miR‐210‐fold change (FC) values were extracted directly. Where relevant data were missing from the text but available in figures, the WebPlotDigitizer version 4 tool^29^ was used to extract information from these visual representations. In the case of the study by Biro et al.,^30^ we obtained individual‐level data that enabled us to perform direct outcome calculations. For studies in which data on miR levels were not reported, the corresponding authors were contacted via email to request additional information; however, no additional responses were obtained. The following variables were extracted from the eligible articles: first author, year of publication, study type and design, country, characteristics of study participants, severity of PE (mild or severe), if present “early‐onset” or “late‐onset” forms of PE, gestational age at sample collection (on a scale of weeks or trimesters), birth weight and gestational age at termination of pregnancy, methods used to assess expression, normalization methods, relative differences in miR‐210 expression levels between groups (fold changes in mean or median values), and standard deviation (SD)/standard error (SE) or minimum and maximum values of the outcome.

### 2.6 Assessment of risk of bias

Two authors (D.M. and Zs.E.) independently assessed the risk of bias, with a third reviewer (A.V.) resolving any disputes. Eligible articles were evaluated using the Joanna Briggs Institute (JBI) Critical Appraisal Checklists.^31^ The VISualization (Robvis) tool^32^ was employed for visualization, with minor modifications to align with the JBI checklist.

### 2.7 Data synthesis

Criteria for statistical poolability required at least three clinically comparable articles with the aligned research questions reporting the same outcome. Studies reported point estimates and measures of spread for the relative increase in miR‐210 expression in patients with PE compared to healthy controls. Means and SDs were estimated in cases where quartiles were reported. Precalculated effect sizes were pooled to obtain a weighted mean and 95% confidence interval (CI) for the fold‐increase in miR‐210 expression. Subgroup analyses were performed according to the severity of PE, categorized as “mild” and “severe.” We assumed different τ^2^ values in the subgroups. A multivariate approach was used with PE severity as a moderator and the study as a random effect to address within‐study correlation as common controls were applied for the subgroups. To explore heterogeneity and the influence of studies, we performed a leave‐one‐out analysis, where one article was excluded, and the model was refitted. Additionally, where studies reported effect sizes for both mild and severe groups, we calculated the ratio of fold increases between the subgroups. Lack of independence due to common controls was handled by implementing correlation when calculating the SD of the ratio of fold increases, using the delta method. An inverse variance method was employed to synthesize effect sizes across studies in random‐effects meta‐analysis models. The restricted maximum likelihood estimator was used to derive the between‐study variance (tau^2^).

Forest plots were utilized to graphically represent the summarized results. Where appropriate, prediction intervals were provided to indicate the expected variability of effects in future studies. Heterogeneity among studies was assessed and described using the *I* ^2^ statistic as defined by Higgins and Thompson.^33^ The guidelines of Harrer et al. were followed for theoretical considerations.^34^ Practical implementation was conducted in R^35^ using the meta^36^ and metafor^37^ packages for basic meta‐analysis calculations, plots, and estmeansd^38^ to estimate means and medians from quartiles. Due to insufficient available data, subgroup analysis based on sampling timing and comparison between early‐onset and late‐onset PE groups could not be performed.

## 3 RESULTS

### 3.1 Study selection

A total of 776 studies were identified through a systematic search of five databases. After duplicate removal, 300 records were analyzed by title and abstract. During the full‐text selection, 32 eligible articles were screened. No studies were found eligible through the citation chaser.^25^ Reasons for exclusion during the full‐text selection process are detailed in Table S1. One non‐English full‐text article written in Russian was identified during the selection process and translated using Google Translate for eligibility assessment. After evaluation of the translated full text, the article did not meet the inclusion criteria and was excluded. Consequently, no non‐English studies were included in the synthesis, and no data from non‐English articles were extracted. Ultimately, seven articles were deemed suitable for quantitative and qualitative synthesis, whereas two were included for qualitative synthesis only (Figure 1).

**FIGURE 1:**
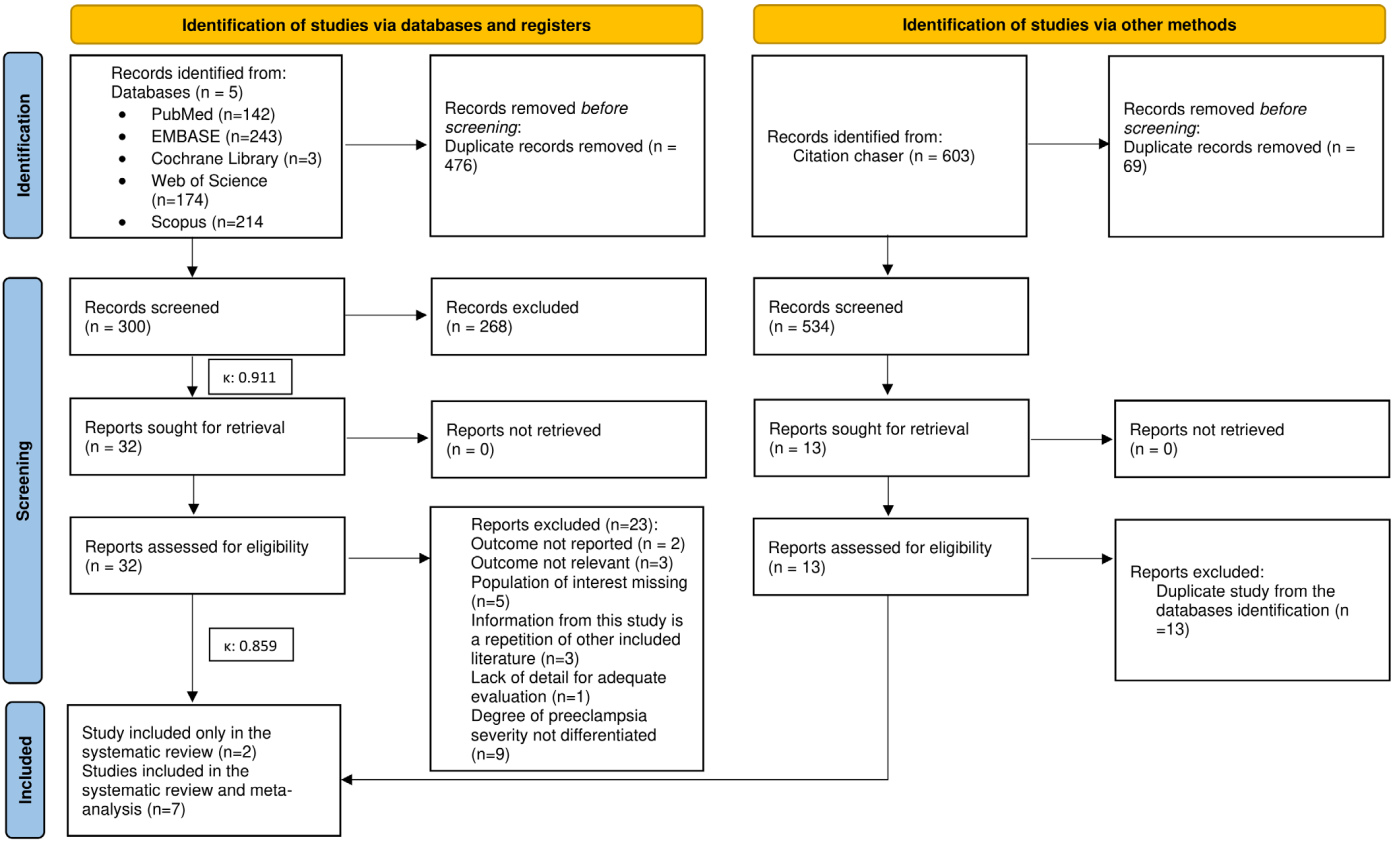
Preferred Reporting Items for Systematic reviews and Meta‐Analyses (PRISMA) 2020 flow chart representing the study selection process.

### 3.2 Study characteristics

The eligible articles were published between 2012 and 2021. Our systematic review and meta‐analysis included 75 patients with mild PE, 110 patients with severe PE, and 157 healthy controls. Blood pressure values from the different groups are presented and aligned with disease definitions. Six studies were two‐armed case–control studies comparing groups with mild and severe PE. MiR‐210 levels were sampled primarily in the second and third trimesters. All studies utilized plasma samples for analysis, the majority of which used quantitative reverse transcription polymerase chain reaction (qRT‐PCR) for miR analysis; only one study used droplet digital polymerase chain reaction (ddPCR).^39^ Three different normalization controls were used to quantify miR expression levels: Cel‐miR‐39,^39^, ^40^, ^41^, ^42^ U6,^43^, ^44^, ^45^ and hsa‐miR‐103a.^30^, ^46^ Table 1 details the baseline characteristics of the enrolled studies, while Table S3 summarizes the basic maternal and fetal characteristics.

**TABLE 1:** Basic characteristics of included studies.

Author (year)	Region	Number of patients			Systolic BP^a^ (mmHg)			Diastolic BP^a^ (mmHg)			Gestational week at sampling	Normalization control	Method used for miR analysis
		Control	Mild PE	Severe PE	Control	Mild PE	Severe PE	Control	Mild PE	Severe PE			
Zhang et al. (2012)^43^	China	15	15	15	112 ± 6.3	151 ± 7.3	173 ± 7.8	72 ± 5.7	98 ± 4.7	109 ± 7.8	28–40	U6	qRT‐PCR
Ura et al. (2014)^40^	Italy	24	NR	24	NR	NR	NR	NR	NR	NR	12–14	Cel‐miR‐39	qRT‐PCR
Murphy et al. (2015)^41^	Canada	17	7	6	NR	NR	NR	NR	NR	NR	36–40	Cel‐miR‐39	qRT‐PCR
Jairajpuri et al. (2017)^42^ ^,^ ^b^	Bahrain	7	7	8	108 (90–120)	144 (140–152)	170 (165–180)	70 (60–80)	92.5 (90–95)	115 (110–120)	28–40	Cel‐miR‐39	qRT‐PCR
Biro et al. (2017)^30^	Hungary	34	15	19	NR	NR	NR	NR	NR	NR	22–40	hsa‐miR‐103a	qRT‐PCR
Youssef et al. (2019)^44^	Egypt	20	12	18	114 ± 6.6	145 ± 5.2	173 ± 16.4	69.5 ± 9.3	92.5 ± 4.5	113 ± 7.6	38–40	U6	qRT‐PCR
Jelena et al. (2020)^39^ ^,^ ^b^	Serbia	17	19	NR	110 ± 14	138 ± 15	NR	71 ± 11	89 ± 13	NR	20–38	Cel‐miR‐39	ddPCR
Jairajpuri et al. (2021)^45^	Bahrain	15	15	15	105 (90–120)	143 (140–146)	172 (165–179)	71 (65–77)	96 (90–102)	112 (95–129)	28–40	U6	qRT‐PCR
Kolkova et al. (2021)^46^	Slovakia	32	11	13	128 (99–147)	144 (125–160)	164 (134–198)	79 (54–91)	96 (75–106)	106 (84–120)	NR	hsa‐miR‐103a	qRT‐PCR

Abbreviations: BP, blood pressure; ddPCR, droplet digital polymerase chain reaction; MiR: microRNA; mPE, mild pre‐eclampsia; NR, not reported; PE, pre‐eclampsia; SD, standard deviation; sPE, severe pre‐eclampsia; qRT‐PCR, quantitative reverse transcription polymerase chain reaction.

^a^ mmHg–parameters represented as mean with SD, or median with range (minimum and maximum).

^b^ Study included only in the systematic review.

### 3.3 Risk of bias of included studies

Our analysis indicated that the most significant issue was the lack of strategies to address confounding factors. Additionally, most studies did not document the appropriate matching of the groups compared. Only one study utilized an alternative miR quantification tool that differed from qRT‐PCR, the current gold standard for analysis.^39^ The results of the risk of bias assessment are presented in Figure 2.

**FIGURE 2:**
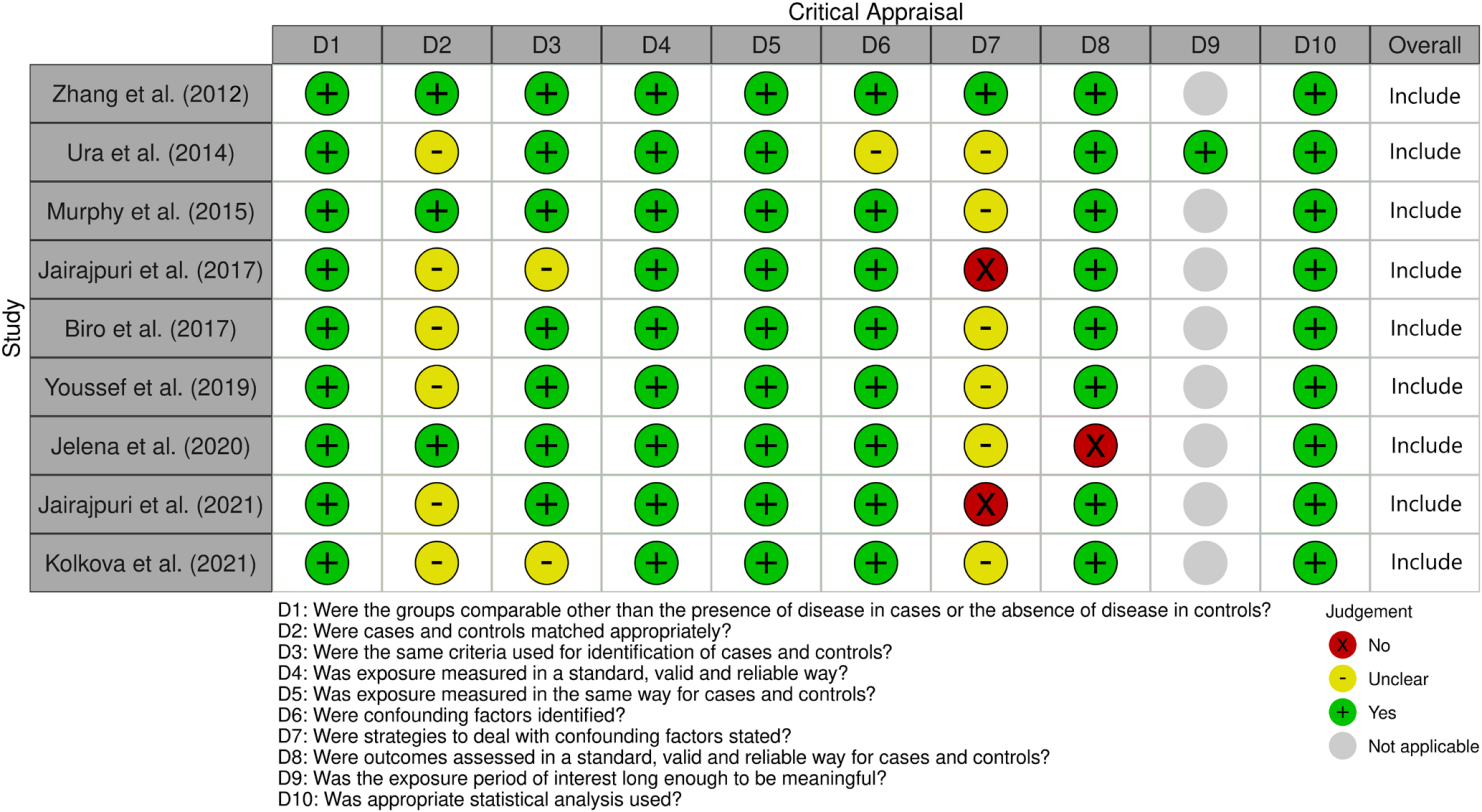
Joanna Briggs Institute (JBI) quality checklist for risk of bias analysis of the selected articles. Summary of the risk of bias evaluation for each included study using the JBI quality checklist. Green circles denote a “Yes” judgment, indicating a low risk of bias; yellow circles represent an unclear risk; and red circles signify a “No” judgment, indicating a high risk of bias. Key sources of bias include a lack of confounder control and group mismatching.

### 3.4 Synthesis of results

#### 3.4.1 MicroRNA‐210 levels across degrees of PE severity in pregnant women

Quantitative synthesis was feasible for articles that provided variability parameters for outcomes, either directly or indirectly. Six case–control studies involving 133 controls and 75 patients with PE were analyzed in the mild subgroup.^30^, ^41^, ^43^, ^44^, ^45^, ^46^ The fold change (pooled effect size) (FC) in the mild group was 3.33, with a 95% CI of 0.695.98, indicating a statistically significant difference (*P* = 0.018). The heterogeneity of the studies expressed as *I* ^2^ was 93% (CI: 88–96%), which reflects a substantial variability in the results across the included studies. One outlier study^45^ reported a three‐fold increase in miR‐210 compared to others, contributing to the high heterogeneity. A ddPCR study for miR assessment found no significant difference between the control group and the group with mild PE.^39^ As variability values could not be calculated for this study, it was included only in the qualitative analysis. In the severe subgroup,^30^, ^40^, ^41^, ^43^, ^44^, ^45^, ^46^ seven case–control studies involving 157 controls and 110 patients with PE were analyzed and had a 7.23‐fold change (CI: 2.27–12.18; *I* ^2^ = 98%, CI: 98–99%), with a statistically significant difference within the group (*P* < 0.001). Subgroup difference tests indicated a statistically significant effect (*P* = 0.01) (Figure 3).

**FIGURE 3:**
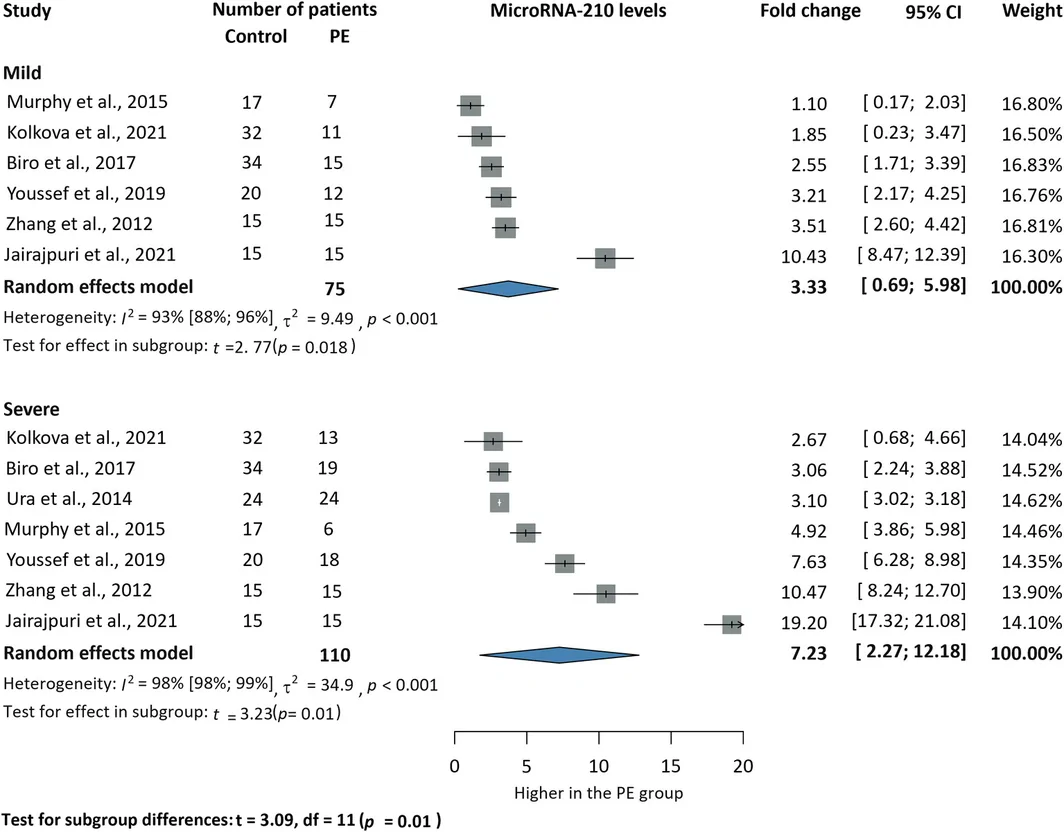
Forest plot representing microRNA‐210 levels across degrees of pre‐eclampsia severity in pregnant women. Fold change: The ratio of microRNA‐210 levels between the pre‐eclamptic and the control groups. The two blue diamonds represent the summary effect estimate from all studies in the mild and severe subgroups. CI, confidence interval; PE, pre‐eclampsia.

Six of the included articles compared populations with both mild and severe PE to a healthy control group (two‐arm studies). The mean ratio of miR‐210‐fold change levels between the two severity groups was 2.05 (CI: 1.41–2.69; *I* ^2^ = 85%; *P* < 0.01), favoring the population with severe PE (Figure 4). Our analysis revealed that miR‐210 expression is more than three‐fold increased in mild PE and more than seven‐fold increased in severe cases compared to healthy pregnancies, and that this difference is both clinically and statistically significant. In each individual study, severe values consistently exceeded mild ones, with an average increase of approximately two‐fold. However, we found insufficient data for early‐ and late‐onset PE types to include in our analyses.

**FIGURE 4:**
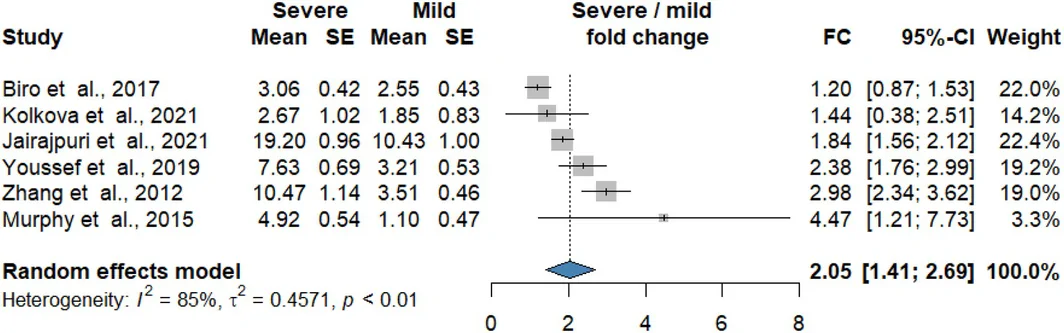
Forest plot representing the association between microRNA‐210 levels and severity of pre‐eclampsia within two‐arm studies. Severe/mild fold change: The ratio of the fold changes in microRNA‐210 levels between the pre‐eclamptic and control groups. A blue diamond at the bottom of the plot represents the summary effect estimate from all two‐armed studies in the meta‐analysis. CI, confidence interval; FC, fold change; SE, standard error.

#### 3.4.2 Results of leave‐one‐out sensitivity analysis

To assess the influence of individual studies on the overall meta‐analysis results, a leave‐one‐out sensitivity analysis was performed. This involved iteratively removing a study at a time and recalculating the pooled effect size. Table S4 summarizes the results of this analysis, presenting the effect size (fold change [FC]) and the confidence interval (CI lower, CI upper) after omitting each study (Table S4).

The analysis revealed that omitting a single study in the mild subgroup did not drastically change the overall effect. The effect size (FC) ranged from 2.25 (omitting Jairajpuri et al., 2021^45^) to 3.76 (omitting Ura et al., 2014^40^). CIs also remained relatively consistent, suggesting that no single study disproportionately affected the pooled estimate in this subgroup. Similarly, in the severe subgroup, the leave‐one‐out analysis indicated that the overall effect was robust to the exclusion of any single study. The effect size ranged from 5.16 (omitting Jairajpuri et al., 2021^45^) to 7.96 (omitting Kolkova et al., 2021^46^). CIs remained relatively stable, further supporting the reliability of the pooled effect size in the severe subgroup (Table S4).

### 3.5 Publication bias and heterogeneity

Table 1 outlines the methodological factors that may account for substantial heterogeneity, including variations in gestational ages at sampling between groups and differences in normalization control methods. Despite substantial heterogeneity between studies (*I* ^2^ = 93% for the mild group and *I* ^2^ = 98% for the severe group), miR‐210 values were consistently elevated when comparing the healthy control population to the pre‐eclamptic group.

Although publication bias could not be formally assessed due to the limited number of studies (*n* < 10), the tendency to publish positive results in miRNA research may have influenced our findings.

## 4 DISCUSSION

In this systematic review and meta‐analysis, we aimed to compare miR‐210 levels between healthy pregnant women and those with PE at different levels of severity, to facilitate research on future biomarkers for this condition.

Given the current limitations in the understanding of PE, the significance of preclinical studies in identifying new predictive approaches should be emphasized. As PE remains incurable, the evaluation and development of predictive methods are essential, mainly as various preventive strategies have been explored.^47^

Our quantitative analysis revealed that miR‐210 expression increased more than three‐fold in mild PE and over seven‐fold in severe cases compared to healthy pregnancies, and this difference is both clinically and statistically significant.

Recent studies have investigated miRs as potential biomarkers, with a meta‐analysis indicating that upregulated circulating miRs serve as more reliable predictors of PE development than downregulated ones.^48^

### 4.1 Comparison with existing literature

A previous meta‐analysis^49^ reported an uncertain relationship between heterogeneous miR‐210 levels and the risk of PE. However, they primarily assessed placental samples and did not address clinical aspects of the disease, such as severity, nor did they incorporate clinically measurable data from plasma samples.^49^

### 4.2 Strengths and limitations

This meta‐analysis is the first to illustrate the potential correlation between elevated levels of circulating miR‐210 and varying degrees of PE severity, thus facilitating further research that may offer several clinical benefits. We adhered to a strict preregistered protocol and employed a rigorous methodology. Our study also included individual data assessments. Given the different definitions of PE in the articles, we made a concerted effort to include only studies that appropriately defined PE and followed standard definitions.

In terms of the limitations of this work, heterogeneity between the included articles and the low number of cases in the sole case–control studies are notable factors. The limited number of eligible studies reflects the stringent eligibility criteria applied, which required guideline‐based diagnostic definitions, circulating rather than placental miR‐210 data, inclusion of both mild and severe PE subgroups, and extractable measures of variability. Despite a comprehensive search of five major databases, supplemented by forward and backward citation chasing, only seven studies met all criteria simultaneously, suggesting that the limited evidence base primarily reflects the scarcity of directly relevant studies rather than a limitation of the search strategy. Differences in the definitions of PE, sampling times, sample storage, and miR assessment techniques contribute to the heterogeneity and represent significant limitations. Differences in PE definitions, sampling timing, sample storage, and miR‐210 assessment techniques contributed to heterogeneity and represented important limitations. The absence of first‐trimester sampling across all included studies, which measured miR‐210 predominantly in the second or third trimester at or near the time of PE diagnosis, limits the scope of our findings to severity stratification at clinical presentation rather than early prediction of PE development or severity before disease onset. Any interpretation of miR‐210 as an antenatal predictive biomarker, therefore, remains speculative and requires dedicated prospective cohort studies with sampling initiated in the first trimester.

Because heterogeneity across the included studies was substantial, calculation of pooled sensitivity, specificity, and positive and negative predictive values for miR‐210 was not feasible. This limitation was further compounded by the absence of receiver operating characteristic data in the included articles, which precluded the derivation of clinically meaningful diagnostic thresholds. Until standardized protocols are adopted and these metrics are consistently reported, the clinical utility of miR‐210 as a standalone biomarker for PE severity cannot be established.

The heterogeneity of PE begins with the fact that studies consider it as a single disease; however, recent cluster‐based classifications, such as the one proposed by Than et al.,^50^ support the notion that it comprises various molecular and clinical subgroups.^50^ Recognition of these distinct subtypes may facilitate the identification of targeted prevention, prediction, and treatment strategies, thereby advancing personalized care.^6^ Enhancing risk stratification enables a more precise identification of individuals who may benefit from current preventive measures, such as aspirin, and future promising medications, such as pravastatin.^13^, ^47^

Another limitation that hindered the feasibility of subgroup analyses was the lack of detailed reporting on plasma miR‐210 levels in early‐onset versus late‐onset PE. None of the included studies provided sufficient data to allow stratification according to disease onset, which restricts interpretation of potential pathophysiological differences between these clinically distinct subtypes.

Additionally, varying definitions of PE contribute to heterogeneity across studies, complicating data synthesis and comparison. Some studies, such as the one by Gunel et al.,^51^ did not publish clinical data, making it challenging to verify the defined population with PE. In other instances, as shown by Munaut et al.,^52^ clinical data did not align with the appropriate PE definition. Standardized definitions of PE and comprehensive publication of patient clinical data and miR outcome parameters, are crucial for achieving more comparable outcomes to guide future research and ultimately improve patient care.

Methodological differences in miR assessment may also contribute to the observed heterogeneity. First, normalization strategies varied substantially across studies. Three reference controls (cel‐miR‐39, U6, and hsa‐miR‐103a) were used, and variability in measured miR‐210 levels was observed even within the same research group, depending on the normalization approach, underscoring the potential impact of this methodological choice on quantitative results. Second, quantification platforms also differed: most studies used qRT‐PCR, whereas one study employed ddPCR, which fundamentally differs in its approach to absolute quantification. Standardization of these methods remains essential but is currently lacking. Methodological variability begins at the preanalytical stage, including sample collection and storage. Most studies used ethylenediaminetetraacetic acid (EDTA) collection tubes, whereas others, including Zhang et al.,^43^ used TRIzol tubes, which may better preserve RNA integrity.^53^ Samples were also handled under different processing and storage conditions, including variable time at room temperature and storage at low temperatures before processing, both of which may influence measured miRNA concentrations.^30^, ^45^ Further variability arises from differences in the miR‐210 fraction analyzed. Whereas most studies measured total exosomal miR‐210, Biro et al.,^54^ specifically examined argonaute‐bound miR‐210 and reported higher levels in this bound form than in the exosomal fraction.^54^ Such differences in sample processing, normalization, quantification platform, and analyte selection may have introduced measurement bias and likely contributed to the substantial between‐study heterogeneity. Future studies should therefore adopt standardized protocols for sample handling, RNA extraction, normalization, quantification, and miR‐210 fraction selection before miR‐210 can be robustly validated as a biomarker of PE severity.

Another key challenge in the analysis of circulating miRs is the lack of validated housekeeping genes. There was notable variability in the increase in miR levels within the same research group sampling a similar population, depending on the normalization tool used (cel‐miR‐39^42^ versus U6^45^).^42^, ^45^ In some cases, internal controls (most commonly U6,^43^, ^44^, ^45^ or hsa‐miR‐103a^30^, ^46^) were used, while in other cases, external controls (cel‐miR‐39^39^, ^40^, ^41^, ^42^) were used to normalize miRs. The use of different housekeeping genes for miRNA normalization can significantly alter the results, representing a major limitation.^55^

It is important to note that, despite substantial between‐study variability in effect magnitude, the direction of effect was consistent across all included studies. MiR‐210 was elevated in pre‐eclamptic women relative to healthy controls, and severe PE values exceeded mild PE values in every individual study. The interpretation of heterogeneity in this context also warrants additional consideration, as the pooled outcome represents a fold change, which is inherently a ratio of means rather than a simple mean. Ideally, such data would be analyzed using a ratio‐of‐means approach requiring separate reporting of control and exposed group values. However, because most included studies reported only precalculated fold changes without disaggregated group‐level data, we were constrained to treat the fold change as a single continuous outcome. This limitation may have contributed to the observed heterogeneity and complicates its interpretation. Therefore, the pooled effect size should be interpreted cautiously as an estimate of magnitude. Nevertheless, the consistent direction of effect across independent research groups, study populations, and methodological settings supports a biologically plausible qualitative association between elevated miR‐210 expression and PE severity. Mestdagh et al.^56^ propose a normalization approach based on mean expression values to address this issue. They recommend creating specific workflows tailored to a limited number of miRNAs, which involve the selection of miRs or small RNA controls with expression profiles that closely resemble the mean expression value.^56^

A significant challenge impacting performance is the cost. Incorporating more controls and using more expensive tools for miR assessment will inevitably increase overall expenses, complicating clinical usability. Therefore, it is essential to develop cost‐effective, high‐throughput assays to integrate miR assessment into routine obstetric care.^57^

### 4.3 Implications

Our findings hold great promise for future research, directing attention to the measurement of miR‐210 levels in PE. Our study shows the association between the pathogenesis of PE and miR‐210.

MiR‐210 may serve as a prognostic marker for severity at the time of the diagnosis of PE. Realizing this potential requires well‐designed prospective cohort studies that employ standardized miR assessment technologies to establish clinically valuable prediction intervals for severity.

One of the most important biomarkers currently known for PE the soluble fms‐like tyrosine kinase‐1/placental growth factor ratio (sFlt‐1/PlGF ratio) has primarily been investigated for its predictive accuracy and has been shown to provide a high negative predictive value for pregnancy outcomes in high‐risk patients.^58^

As the sFlt‐1/PlGF ratio has demonstrated strong negative predictive value for adverse pregnancy outcomes, miR‐210 may warrant investigation as a complementary marker, particularly given its consistent elevation in severe PE across the included studies.^58^, ^59^ However, this proposed complementarity remains an untested hypothesis, and prospective studies directly comparing and combining these markers are required before any added clinical value over existing biomarkers can be substantiated.

To evaluate the potential predictive value of miR‐210 for PE, prospective cohort studies with large sample sizes from early pregnancy are needed, comparing plasma profiles from women who develop PE with those who remain normotensive.

The translation of scientific findings into clinical practice is of great significance,^60^, ^61^ and could be integrated into the prognostic assessment of PE once its negative and positive predictive values are established.

## 5 CONCLUSION

This systematic review and meta‐analysis demonstrates that circulating miR‐210 levels are strongly associated with PE severity at the time of diagnosis, with a consistent, significant stepwise increase from mild to severe disease. These findings support a potential role for miR‐210 as a biomarker of disease severity rather than as an established early predictive marker.

## AUTHOR CONTRIBUTIONS

**D**.**M**.: Conceptualization, project administration, methodology, formal analysis, and writing – original draft. **A**.**V**.: Conceptualization and data curation. **Zs**.**E**.: Conceptualization, data curation. **I**.**P**.**A**.**V**.: Conceptualization, project administration, supervision, methodology, writing – review and editing. **P**.**H**.: Conceptualization, project administration, and methodology. **M**.**O**.: Conceptualization, methodology, writing – review and editing. **M**.**B**.: conceptualization, and data analysis. **Z**.**M**.: conceptualization, and supervision. **S**.**V**.: Conceptualization, supervision, writing – review and editing. All authors certify that they have participated sufficiently in the work to take public responsibility for the content, including participation in the concept, design, analysis, writing, or revision of the manuscript.

## FUNDING INFORMATION

Open access funding was provided by Semmelweis University. Sponsors had no role in the design, data collection, analysis, interpretation, and manuscript preparation. IPAV was supported by the EKÖP‐2024 scholarships–the New National Excellence Program of the Ministry of Culture and Innovation from the source of the National Research, Development and Innovation Office fund. The funding agency had no role in the design, data collection, analysis, interpretation, and manuscript preparation.

## CONFLICT OF INTEREST STATEMENT

The authors have nothing to declare.

## Supporting information


Table S1.
Table S2:
Table S3:
**Table S4**.

Data S1.

## Data Availability

Research data are not shared.
