# Supplementary material for: MicroRNA‐210 level as a biomarker for pre‐eclampsia severity: A systematic review and meta‐analysis
Source: Int J Gynaecol Obstet. 2026 Jun 23;175(3):1177–87. doi: 10.1002/ijgo.71145 (PMC13769348; doi:10.1002/ijgo.71145)
Supplement: Supplementary file 2 [file IJGO-175-1177-s002.docx]

**Supplementary Material**

**Title**

**MicroRNA-210 Level as a Biomarker for Preeclampsia Severity: A Systematic Review and Meta-Analysis**

**Authors**

Dávid Márai^1,2^, Isabel Pinto Amorim das Virgens^1^, Ádám Vincze^1,2^, Mahmoud Obeidat^1^, Péter Hegyi^1,3,4^, Mikolt Bakony^1^, Zsófia Réka Erdélyi^1,2^, Zsolt Melczer^1,2^, Sándor Valent^1,2^

**Affiliations:**

1. Centre for Translational Medicine, Semmelweis University, Budapest, Hungary
2. Department of Obstetrics and Gynecology, Semmelweis University, 1088 Budapest, Hungary
3. Institute for Translational Medicine, Medical School, University of Pécs, Pécs, Hungary
4. Institute of Pancreatic Diseases, Semmelweis University, 1088 Budapest, Hungary

**Corresponding author**

Sándor Valent MD, Phd

Postal address: H-1082 Üllői út 78/A., Budapest, Hungary

Tel.: +36 1 459-1500; E-mail address: valent.sandor@semmelweis.hu

**Figure and table legends**

**1. Supplementary Method**

**1.1 Supplementary Table S1.** - Reason for exclusion in the full-text selection

**1.2 Supplementary Table S2.** - PRISMA 2020 checklist ^1^

**1.3 Search Key**

**1.4 MOOSE checklist ^2^**

**2. Supplementary Material**

**2.1 Supplementary Table S3. Basic maternal and fetal characteristics in the included studies**

Parameters represented as a mean with standard deviation, or median with range (minimum and maximum),

‡ Body mass index (kilogram/meter2)

† Study included only in the systematic review

* - Data from the total preeclampsia group (including mild and severe subgroups) presented

C: control group; mPE: mild preeclampsia; sePE: severe preeclampsia; PE: Preeclampsia; NR: Not Reported; miR: microRNA

**2.2 Supplementary Table S4.** Leave-one-out analysis of the included studies

This table presents the results of the leave-one-out meta-analysis, showing the effect (FC- fold change) of omitting each study on the overall effect size and confidence intervals (CI lower and upper values).

**3. References**

**1. Supplementary Method**

**1.1 Supplementary Table S1. -** Reason for exclusion in the full-text selection

| **Name of the study** | **Reason for exclusion** | **Additional comments** |
| --- | --- | --- |
| Gunel et al. (2011) ^3^ | Outcome is not relevant | The microRNA-210 values are reported only as a ratio compared to the microRNA-153 levels. |
| Elovitz et al. (2012) ^4^ | Information from this study is a repetition of other included literature |  |
| Elovitz et al. (2013) ^5^ | Information from this study is a repetition of other included literature |  |
| Anton et al. (2013) ^6^ | Population of interest is missing | No separated preeclamptic group; only an “HDP - Hypertensive Disorder of Pregnancy” mixed population was observed. |
| Winger et al (2015) ^7^ | Outcome is not relevant | Measures differences in the microRNA response to intravenous immunoglobulin therapy between preeclampsia and healthy pregnancies. The degree of preeclampsia severity is not differentiated. |
| Li et al. (2015) ^8^ | Degree of preeclampsia severity is not differentiated | General preeclampsia population with mild and severe preeclamptic cases mixed. |
| Hromadnikova et al. (2016) ^9^ | Outcome is not reported | Data about microRNA-210 is not reported. |
| Munaut et al. (2016) ^10^ | Population of interest is missing according to the clinical characteristics table. Degree of preeclampsia severity is not differentiated. | The preeclamptic group contains normotensive patients. Significantly higher microRNA-210 levels in the preeclamptic group compared to the control population. |
| Gan et al. (2017) ^11^ | Degree of preeclampsia severity is not differentiated | General preeclampsia population with mild and severe preeclamptic cases mixed. The mixed population had a 1,37 significant fold change in microRNA-210 levels compared to the control population. |
| Han et al. (2018) ^12^ | Population of interest is missing | The study compared two preeclamptic populations based on the pregnancy outcome. |
| Biró et al. (2019) ^13^ | Degree of preeclampsia severity is not differentiated | General preeclampsia population with mild and severe preeclamptic cases mixed. The outcome parameters are also not generalizable as they were measured from different parts of the serum samples |
| Nejad et al. (2019) ^14^ | Degree of preeclampsia severity is not differentiated | General preeclampsia population with mild and severe preeclamptic cases mixed. The mixed population (mainly mild preeclamptic but not clearly defined) had a 3,34 significant fold change in microRNA-210 levels compared to the control population. |
| Akgör et al. (2020) ^15^ | Degree of preeclampsia severity is not differentiated. | General preeclampsia population is mixed with mild and severe preeclamptic cases. The mixed population had a 7,28 significant fold change in microRNA-210 levels compared to the control population. |
| Ghafari et al. (2020) ^16^ | Lack of detail for adequate evaluation |  |
| Lin et al. (2020) ^17^ | Degree of preeclampsia severity is not differentiated | General preeclampsia population is mixed with mild and severe preeclamptic cases. The mixed population has significantly higher microRNA-210 levels compared to the control population. Exact microRNA values are not reported |
| Trongpisutsak et al. (2021) ^18^ | Population of interest is missing according to the clinical characteristics table.  The degree of preeclampsia severity is not differentiated | The preeclamptic group is normotensive. |
| Abbas et al. (2021) ^19^ | Information from this study is a repetition of other included literature |  |
| Jin et al. (2021) ^20^ | Population of interest is missing | Patients with “hypertensive disorder complicating pregnancy (HDCP),” microRNA-210 values are compared to each other, not to the healthy control population |
| Pashkovszky et al. (2023) ^21^ | Outcome not reported | MicroRNA-210 values are not reported |
| Soobryan et al. (2023) ^22^ | Degree of preeclampsia severity is not differentiated. | Only early-onset and late-onset forms are reported |
| Bowman-Gibson et al. (2024) ^23^ | Outcome is not relevant | The outcome parameters are not generalizable as they were measured from different parts of the serum samples. |
| Hasdemir et al. (2024) ^24^ | Degree of preeclampsia severity is not differentiated |  |
| Ozler et al. (2024) ^25^ | Degree of preeclampsia severity is not differentiated | Significantly higher microRNA-210 levels in the preeclamptic group compared to the control population. |

**1.2 Supplementary Table S2.** – PRISMA 2020 checklist ^1^

| **Section and Topic** | **Item #** | **Checklist item** | **Location where item is reported** |
| --- | --- | --- | --- |
| **TITLE** | | |  |
| Title | 1 | Identify the report as a systematic review. | 1 |
| **ABSTRACT** | | |  |
| Abstract | 2 | See the PRISMA 2020 for Abstracts checklist. | 5 |
| **INTRODUCTION** | | |  |
| Rationale | 3 | Describe the rationale for the review in the context of existing knowledge. | 7 |
| Objectives | 4 | Provide an explicit statement of the objective(s) or question(s) the review addresses. | 8 |
| **METHODS** | | |  |
| Eligibility criteria | 5 | Specify the inclusion and exclusion criteria for the review and how studies were grouped for the syntheses. | 9 |
| Information sources | 6 | Specify all databases, registers, websites, organisations, reference lists and other sources searched or consulted to identify studies. Specify the date when each source was last searched or consulted. | 9 |
| Search strategy | 7 | Present the full search strategies for all databases, registers and websites, including any filters and limits used. | Suppl. Material |
| Selection process | 8 | Specify the methods used to decide whether a study met the inclusion criteria of the review, including how many reviewers screened each record and each report retrieved, whether they worked independently, and if applicable, details of automation tools used in the process. | 10 |
| Data collection process | 9 | Specify the methods used to collect data from reports, including how many reviewers collected data from each report, whether they worked independently, any processes for obtaining or confirming data from study investigators, and if applicable, details of automation tools used in the process. | 10 |
| Data items | 10a | List and define all outcomes for which data were sought. Specify whether all results that were compatible with each outcome domain in each study were sought (e.g. for all measures, time points, analyses), and if not, the methods used to decide which results to collect. | 11 |
|  | 10b | List and define all other variables for which data were sought (e.g. participant and intervention characteristics, funding sources). Describe any assumptions made about any missing or unclear information. | 11 |
| Study risk of bias assessment | 11 | Specify the methods used to assess risk of bias in the included studies, including details of the tool(s) used, how many reviewers assessed each study and whether they worked independently, and if applicable, details of automation tools used in the process. | 11 |
| Effect measures | 12 | Specify for each outcome the effect measure(s) (e.g. risk ratio, mean difference) used in the synthesis or presentation of results. | 11 |
| Synthesis methods | 13a | Describe the processes used to decide which studies were eligible for each synthesis (e.g. tabulating the study intervention characteristics and comparing against the planned groups for each synthesis (item #5)). | 12 |
|  | 13b | Describe any methods required to prepare the data for presentation or synthesis, such as handling of missing summary statistics, or data conversions. | 12 |
|  | 13c | Describe any methods used to tabulate or visually display results of individual studies and syntheses. | 12 |
|  | 13d | Describe any methods used to synthesize results and provide a rationale for the choice(s). If meta-analysis was performed, describe the model(s), method(s) to identify the presence and extent of statistical heterogeneity, and software package(s) used. | 12 |
|  | 13e | Describe any methods used to explore possible causes of heterogeneity among study results (e.g. subgroup analysis, meta-regression). | 12 |
|  | 13f | Describe any sensitivity analyses conducted to assess robustness of the synthesized results. | 11 |
| Reporting bias assessment | 14 | Describe any methods used to assess risk of bias due to missing results in a synthesis (arising from reporting biases). | 11 |
| Certainty assessment | 15 | Describe any methods used to assess certainty (or confidence) in the body of evidence for an outcome. | 12 |
| **RESULTS** | | |  |
| Study selection | 16a | Describe the results of the search and selection process, from the number of records identified in the search to the number of studies included in the review, ideally using a flow diagram. | Figure 1. |
|  | 16b | Cite studies that might appear to meet the inclusion criteria, but which were excluded, and explain why they were excluded. | Figure 1.  Suppl. Material |
| Study characteristics | 17 | Cite each included study and present its characteristics. | Table 1. |
| Risk of bias in studies | 18 | Present assessments of risk of bias for each included study. | Figure 2 |
| Results of individual studies | 19 | For all outcomes, present, for each study: (a) summary statistics for each group (where appropriate) and (b) an effect estimate and its precision (e.g. confidence/credible interval), ideally using structured tables or plots. | Figure 3 |
| Results of syntheses | 20a | For each synthesis, briefly summarise the characteristics and risk of bias among contributing studies. | Figure 2.  Table 1. |
|  | 20b | Present results of all statistical syntheses conducted. If meta-analysis was done, present for each the summary estimate and its precision (e.g. confidence/credible interval) and measures of statistical heterogeneity. If comparing groups, describe the direction of the effect. | Figure 3.  Figure 4. |
|  | 20c | Present results of all investigations of possible causes of heterogeneity among study results. | 16 |
|  | 20d | Present results of all sensitivity analyses conducted to assess the robustness of the synthesized results. | 15 |
| Reporting biases | 21 | Present assessments of risk of bias due to missing results (arising from reporting biases) for each synthesis assessed. | 15 |
| Certainty of evidence | 22 | Present assessments of certainty (or confidence) in the body of evidence for each outcome assessed. | - |
| **DISCUSSION** | | |  |
| Discussion | 23a | Provide a general interpretation of the results in the context of other evidence. | 17 |
|  | 23b | Discuss any limitations of the evidence included in the review. | 19 |
|  | 23c | Discuss any limitations of the review processes used. | 19 |
|  | 23d | Discuss implications of the results for practice, policy, and future research. | 20 |
| **OTHER INFORMATION** | | |  |
| Registration and protocol | 24a | Provide registration information for the review, including register name and registration number, or state that the review was not registered. | 1 |
|  | 24b | Indicate where the review protocol can be accessed, or state that a protocol was not prepared. | 1 |
|  | 24c | Describe and explain any amendments to information provided at registration or in the protocol. | 9 |
| Support | 25 | Describe sources of financial or non-financial support for the review, and the role of the funders or sponsors in the review. | 2 |
| Competing interests | 26 | Declare any competing interests of review authors. | 2 |
| Availability of data, code and other materials | 27 | Report which of the following are publicly available and where they can be found: template data collection forms; data extracted from included studies; data used for all analyses; analytic code; any other materials used in the review. | - |

**1.3 Search Key**

(pregn* OR preeclamp* OR pre-eclamp*) AND (”micro-RNA-210” OR ”micro RNA-210” OR ”microRNA-210” OR ”micro-RNA 210” OR ”miRNA-210” OR ”miRNA 210” OR ”miR-210” OR ”miR 210” OR ”miR210”)

Domain 1 refers to the Population – pregnant and preeclamptic women – both are required to find all the key articles. Many articles do not write “pregnant women” in their titles and abstracts, as all preeclamptic women are fundamentally pregnant too.

The truncations “**pregn*”, “preeclamp*” and “pre-eclamp*”** were used to find all varieties of the population, all possible terms that are being used (for example. pregnant, pregnancy, preeclampsia, preeclamptic, pre-eclamptic, preeclampsia, etc.).

Domain 2 refers to Factor – Adding more types of abbreviations does not increase the number of results.

**1.4 MOOSE Checklist ^2^**

| **Reporting Criteria** | **Reported (Yes/No)** | **Reported on Page No.** |
| --- | --- | --- |
| **Reporting of Background** |  |  |
| Problem definition | Yes | 3 |
| Hypothesis statement | Yes | 4 |
| Description of Study Outcome(s) | Yes | 4 |
| Type of exposure or intervention used | Yes | 3 |
| Type of study design used | Yes | 3 |
| Study population | Yes | 3 |
| **Reporting of Search Strategy** |  |  |
| Qualifications of searchers (eg, librarians and investigators) | Yes | 1 |
| Search strategy, including time period included in the synthesis and keywords | Yes | 5 |
| Effort to include all available studies, including contact with authors | Yes | 6 |
| Databases and registries searched | Yes | 5 |
| Search software used, name and version, including special features used  (eg, explosion) | Yes | 5 |
| Use of hand searching (eg, reference lists of obtained articles) | Yes | 5 |
| List of citations located and those excluded, including justification | Yes | 6 |
| Method for addressing articles published in languages other than  English | Yes | 5 |
| Method of handling abstracts and unpublished studies | Yes | 5 |
| Description of any contact with authors | Yes | 6 |
| **Reporting of Methods** |  |  |
| Description of relevance or  appropriateness of studies assembled for assessing the hypothesis to be tested | Yes | 5 |
| Rationale for the selection and coding of data (eg, sound clinical principles or convenience) | Yes | 6 |
| Documentation of how data were classified and coded (eg, multiple raters, blinding, and interrater reliability) | Yes | 6 |
| Assessment of confounding (eg, comparability of cases and controls in studies where appropriate | Yes | 6 |
| **Reporting Criteria** | **Reported (Yes/No)** | **Reported on Page No.** |
| Assessment of study quality, including blinding of quality assessors; stratification or regression on possible predictors of study results | Yes | 6 |
| Assessment of heterogeneity | Yes | 10 |
| Description of statistical methods (eg, complete description of fixed or random effects models, justification of whether the chosen models account for predictors of study results, dose-response models, or cumulative meta-analysis) in sufficient detail to be replicated | Yes | 7 |
| Provision of appropriate tables and graphics | Yes | 9 |
| **Reporting of Results** |  |  |
| Table giving descriptive information for each study included | Yes | 8 |
| Results of sensitivity testing (eg, subgroup analysis) | Yes | 9 |
| Indication of statistical uncertainty of findings | Yes | 10 |
| **Reporting of Discussion** |  |  |
| Quantitative assessment of bias (eg, publication bias) | Yes | 12 |
| Justification for exclusion (eg, exclusion of non–English-language citations) | Yes | Supplement |
| Assessment of quality of included studies | Yes | 11 |
| **Reporting of Conclusions** |  |  |
| Consideration of alternative explanations for observed results | Yes | 13 |
| Generalization of the conclusions (ie, appropriate for the data presented and within the domain of the literature review) | Yes | 13 |
| Guidelines for future research | Yes | 14 |
| Disclosure of funding source | Yes | 15 |

**Supplementary Table S3. -** Basic maternal and fetal characteristics in the included studies

| **Author (year)** | **Region** | **Maternal age (years)** | | | **BMI at miR sampling ‡** | | | **Maternal proteinuria (grams/ 24 hours)** | | | **Fetal gestational age at birth (weeks of gestation)** | | | **Fetal birth weight (grams)** | | |
| --- | --- | --- | --- | --- | --- | --- | --- | --- | --- | --- | --- | --- | --- | --- | --- | --- |
|  |  | **C** | **mPE** | **sePE** | **C** | **mPE** | **sePE** | **C** | **mPE** | **sePE** | **C** | **mPE** | **sePE** | **C** | **mPE** | **sePE** |
| Zhang et al. (2012) ^26^ | China | 29.7 ±3.6 | 31.6 ±3.6 | 30,9 ±4.1 | 25.4 ±5.2 | 26.1 ±4.3 | 25.7 ±4.2 | NR | 0.9 ±0.4 | 3.2 ±1.5 | NR | 33.7 ±3.0 | 32.3 ±3.1 | NR | NR | NR |
| Ura et al. (2014) ^27^ | Italy | 33.7 (30.3-36.1) | NR | 34.4 (33-36.8) | NR | NR | NR | NR | NR | NR | 36.7 (35.1-39) | NR | 40.6 (39.3-41.1) | NR | NR | NR |
| Murphy et al. (2015) ^28^ | Canada | 28.2± 4.1 | 32.4 ±6.9 | 28 ±7.6 | 25.2 ±3.5 | 32.1 ±8.3 | 25.1 ±5.1 | NR | NR | NR | 40.2 ±1.5 | 38 ±1.3 | 35,9 ±3.6 | 3623 ±351 | 3332 ±809 | 2538 ±1023 |
| Jairajpuri et al. (2017) †^29^ | Bahrain | 29 (23-36) | 30 (25-38) | 34 (28-39) | NR | NR | NR | NR | 2.4 (2.1-2.6) | 3.3 (3-3.4) | 38 (37-39) | 33 (29-36) | 31 (27-33) | 3620 (2600-4100) | 2810 (2400-3400) | 2210 (1900-2600) |
| Biro et al. (2017) ^30^ | Hungary | 31.3 (23-38) | 30.8 (20-41) | 30.4 (15-41) | 26.97 (21.3-35.82) | 33.26 (21.72-42.53) | 30.11 (21.56-39.79) | NR | NR | NR | 38.94 (36.14-40.86) | 36.63 (29-40) | 31.12 (22.42-38.86) | 3525 (2160-4770) | 2649 (990-3510) | 1556 (320-3350) |
| Youssef et al. (2019) ^31^ | Egypt | 29.75± 4.24 | 31.8±3.16* | | NR | NR | NR | NR | NR | NR | 38.6±0.94 | 37.75 ±1.35 | 35 ±4.02 | 3480 ±308 | 2975 ±170 | 2448 ±549 |
| Jelena et al. (2020) †^32^ | Serbia | 32 (22-40) | 34 (20-51) | NR | 26 ±3 | 29 ±6 | NR | <0.15 | 0.5 (0.3-4.17) | NR | NR | NR | NR | NR | NR | NR |
| Jairajpuri et al. (2021) ^33^ | Bahrain | 30 (25-35) | 32 (29-35) | 33 (29-37) | NR | NR | NR | NR | 2.2 (1.8-2.6) | 3.4 (3-3.8) | 39 (37-41) | 32 (29-35) | 30 (27-33) | 3300 (3100-3500) | 3200 (2900-3500) | 2400 (2100-2700) |
| Kolkova et al. (2021) ^34^ | Slovakia | 30 (25-37) | 24.5 (21-32) | 29 (22-50) | 26.5 (20.4-40.4) | 29.1 (20.2-44.6) | 31.5 (24.6- 41.6) | None | 1.41 (0.3-3.66) | 1.8 (0.13-9.86) | 40 (37-42) | 38 (27-42) | 37 (28-41) | 3535 (2630-4540) | 2440 (590-3900) | 2930 (660-4290) |

**2.2 Supplementary Table S4. -** Leave-one-out analysis of the included studies

| **Omitted study** | **Subgroup** | **FC** | **CI lower** | **CI upper** |
| --- | --- | --- | --- | --- |
| Omitting Biro et al., 2017 ^30^ | Mild | 3,18 | 0,24 | 6,12 |
| Omitting Jairajpuri et al., 2021 ^33^ | Mild | 2,25 | 1,45 | 3,06 |
| Omitting Kolkova et al., 2021 ^34^ | Mild | 3,32 | 0,45 | 6,21 |
| Omitting Murphy et al., 2015 ^28^ | Mild | 3,59 | 0,92 | 6,25 |
| Omitting Ura et al., 2014 ^27^ | Mild | 3,76 | 1,06 | 6,44 |
| Omitting Youssef et al., 2019 ^31^ | Mild | 3,22 | 0,42 | 1,95 |
| Omitting Zhang et al., 2012 ^26^ | Mild | 3,22 | 0,49 | 5,96 |
| Omitting Biro et al., 2017 ^30^ | Severe | 7,94 | 2,98 | 12,91 |
| Omitting Jairajpuri et al., 2021 ^33^ | Severe | 5,16 | 2,84 | 7,48 |
| Omitting Kolkova et al., 2021 ^34^ | Severe | 7,96 | 3,05 | 12,87 |
| Omitting Murphy et al., 2015 ^28^ | Severe | 7,62 | 2,48 | 12,77 |
| Omitting Ura et al., 2014 ^27^ | Severe | 7,92 | 2,97 | 12,89 |
| Omitting Youssef et al., 2019 ^31^ | Severe | 7,16 | 1,95 | 12,38 |
| Omitting Zhang et al., 2012 ^26^ | Severe | 6,72 | 1,68 | 11,76 |

**3. References**

1.	Page M.J. MJE, Bossuyt P.M., Boutron I., Hoffmann T.C., Mulrow C.D., Shamseer L., Tetzlaff J.M., Akl E.A., Brennan S.E., et al. The PRISMA 2020 statement: an updated guideline for reporting systematic reviews. *BMJ 2021;372:n71* 2021. DOI: <https://doi.org/10.1136/bmj.n71>.

2.	Stroup DF, Berlin JA, Morton SC, et al. Meta-analysis of observational studies in epidemiology: a proposal for reporting. Meta-analysis Of Observational Studies in Epidemiology (MOOSE) group. *Jama* 2000; 283: 2008-2012. 2000/05/02. DOI: 10.1001/jama.283.15.2008.

3.	Gunel T, Zeybek YG, Akçakaya P, et al. Serum microRNA expression in pregnancies with preeclampsia. *Genet Mol Res* 2011; 10: 4034-4040. DOI: 10.4238/2011.November.8.5. | RAYYAN-INCLUSION: {"Adam"=>"Included", "David"=>"Included"} | USER-NOTES: {"David"=>["systematic review. nincs pontos poolable miRNA, nincs severe és mild preeclampsia között differentiatio "]}

10.4238/2011.November.8.5.

4.	Elovitz MA, Olarein-George A, Bastek J, et al. 83: MicroRNA 210 is associated and predicts the development of preeclampsia. *American Journal of Obstetrics & Gynecology* 2012; 206: S51-S52. DOI: 10.1016/j.ajog.2011.10.872.

5.	Elovitz M, Anton L, Bastek J, et al. 647: MicroRNA 210 levels in the first and second trimester of pregnancy are accurate predictors of pregnancy related hypertension. *American Journal of Obstetrics & Gynecology* 2013; 208: S273-S274. DOI: 10.1016/j.ajog.2012.10.813.

6.	Anton L, Olarerin-George AO, Schwartz N, et al. miR-210 Inhibits Trophoblast Invasion and Is a Serum Biomarker for Preeclampsia. *The American Journal of Pathology* 2013; 183: 1437-1445. DOI: 10.1016/j.ajpath.2013.07.021.

7.	Winger EE, Reed JL and Ji X. First-trimester maternal cell microRNA is a superior pregnancy marker to immunological testing for predicting adverse pregnancy outcome. *J Reprod Immunol* 2015; 110: 22-35. 2015/05/13. DOI: 10.1016/j.jri.2015.03.005.

8.	Li Q, Long A, Jiang L, et al. Quantification of preeclampsia-related microRNAs in maternal serum. *Biomedical Reports* 2015; 3: 792-796. DOI: 10.3892/br.2015.524.

9.	Hromadnikova I, Kotlabova K, Hympanova L, et al. Gestational hypertension, preeclampsia and intrauterine growth restriction induce dysregulation of cardiovascular and cerebrovascular disease associated microRNAs in maternal whole peripheral blood. *Thromb Res* 2016; 137: 126-140. 2015/12/04. DOI: 10.1016/j.thromres.2015.11.032.

10.	Munaut C, Tebache L, Blacher S, et al. Dysregulated circulating miRNAs in preeclampsia. *Biomedical Reports* 2016; 5: 686-692. DOI: 10.3892/br.2016.779.

11.	Gan L, Liu Z, Wei M, et al. MiR-210 and miR-155 as potential diagnostic markers for pre-eclampsia pregnancies. *Medicine (Baltimore)* 2017; 96: e7515. DOI: 10.1097/MD.0000000000007515. | RAYYAN-INCLUSION: {"Adam"=>"Included", "David"=>"Included"}

10.1097/md.0000000000007515.

12.	Han L, Zhao Y, Jin Z, et al. Correlation of miRNA and VEGF expression with the outcome of early-onset severe preeclampsia in patients receiving expectant treatment. *Int J Clin Exp Pathol* 2018; 11: 2137-2141. 2018/04/01.

13.	Biró O, Fóthi Á, Alasztics B, et al. Circulating exosomal and Argonaute-bound microRNAs in preeclampsia. *Gene* 2019; 692: 138-144. DOI: 10.1016/j.gene.2019.01.012. Epub 2019 Jan 17. | RAYYAN-INCLUSION: {"Adam"=>"Included", "David"=>"Included"} | RAYYAN-LABELS: Resolved conflict | USER-NOTES: {"David"=>["PECO-ban megfelel, beválogatási kritériumoknak is. Systematic review-ban benne lesz, metában nem, hiszen a szérumnak csak egy specifikus részét vizsgálták."]}

10.1016/j.gene.2019.01.012.

14.	Nejad RMA, Saeidi K, Gharbi S, et al. Quantification of circulating miR-517c-3p and miR-210-3p levels in preeclampsia. *Pregnancy Hypertens* 2019; 16: 75-78. DOI: 10.1016/j.preghy.2019.03.004. Epub 2019 Mar 11. | RAYYAN-INCLUSION: {"Adam"=>"Included", "David"=>"Included"}

10.1016/j.preghy.2019.03.004.

15.	Akgör U, Ayaz L and Çayan F. Expression levels of maternal plasma microRNAs in preeclamptic pregnancies. *Journal of Obstetrics and Gynaecology* 2021; 41: 910-914. DOI: 10.1080/01443615.2020.1820465.

16.	Ghafari A, Lessan pezeshki M and Saffari M. P0069MICRO RNA 155, 210, 494, 29B AND 34A EXPRESSION PROFILE IN PREECLAMPSIA AND NORMAL PREGNANCIES. *Nephrology Dialysis Transplantation* 2020; 35. DOI: 10.1093/ndt/gfaa142.P0069.

17.	Lin W, Teng SW, Lin TY, et al. Combinatorial Analysis of Circulating Biomarkers and Maternal Characteristics for Preeclampsia Prediction in the First and Third Trimesters in Asia. *Diagnostics* 2022; 12. DOI: 10.3390/diagnostics12071533.

18.	Trongpisutsak A and Phupong V. Prediction of preeclampsia using a combination of serum micro RNA-210 and uterine artery Doppler ultrasound. *Science progress* 2021; 104: 368504211036856. DOI: 10.1177/00368504211036856.

19.	Abbas AM, Mammdoh YM, El-Moneum OA, et al. 419 Role of second-trimester plasma microRNA-210 levels in prediction of preeclampsia: prospective cohort study. *American Journal of Obstetrics & Gynecology* 2021; 224: S269. DOI: 10.1016/j.ajog.2020.12.440.

20.	Jin Y, Jia T, Wu X, et al. The predictive value of microRNA in early hypertensive disorder complicating pregnancy (HDCP). *American Journal of Translational Research* 2021; 13: 7288-7293.

21.	Pashkovsky D.G. SEV, Rabadanova Ts.R., Gorbunova P.T., Dubovaya A.B., Muslimova E.R., Khoroz E.E., Karabash Z.S., Sorokina L.E. Expression profile of plasma microRNAs and target genes in patients with complicated pregnancy. *Obstetrics, Gynecology and Reproduction* 2023; 17: 231-243.

22.	Soobryan N, Kumar A, Moodley J, et al. The role and expression of pro/antiangiogenic factors and microRNAs in gestational hypertension and pre-eclampsia. *European Journal of Obstetrics and Gynecology and Reproductive Biology* 2023; 290: 38-42. DOI: 10.1016/j.ejogrb.2023.09.012.

23.	Bowman-Gibson S, Chandiramani C, Stone ML, et al. Streamlined Analysis of Maternal Plasma Indicates Small Extracellular Vesicles are Significantly Elevated in Early-Onset Preeclampsia. *Reproductive Sciences* 2024; 31: 2771-2782. DOI: 10.1007/s43032-024-01591-y.

24.	Hasdemir PS, Celikcekic D, Oztatlici M, et al. Placental expressions of Anti-Mullerian hormone/Receptor, vascular endothelial growth factor and related microRNAs in patients with preeclampsia: a case control study. *Biotech Histochem* 2024; 99: 320-329. 2024/08/21. DOI: 10.1080/10520295.2024.2389516.

25.	Ozler S, Kebapcilar A, Ozdemir EM, et al. Are Vascular Endothelium and Angiogenesis Effective MicroRNA Biomarkers Associated with the Prediction of Early-Onset Preeclampsia (EOPE) and Adverse Perinatal Outcomes? *Reproductive Sciences* 2024; 31: 803-810. DOI: 10.1007/s43032-023-01367-w.

26.	Zhang Y, Fei M, Xue G, et al. Elevated levels of hypoxia-inducible microRNA-210 in pre-eclampsia: new insights into molecular mechanisms for the disease. *J Cell Mol Med* 2012; 16: 249-259. 2011/03/11. DOI: 10.1111/j.1582-4934.2011.01291.x.

27.	Ura B, Feriotto G, Monasta L, et al. Potential role of circulating microRNAs as early markers of preeclampsia. *Taiwanese Journal of Obstetrics and Gynecology* 2014; 53: 232-234. DOI: 10.1016/j.tjog.2014.03.001.

28.	Murphy MS, Casselman RC, Tayade C, et al. Differential expression of plasma microRNA in preeclamptic patients at delivery and 1 year postpartum. *Am J Obstet Gynecol* 2015; 213: 367.e361-369. DOI: 10.1016/j.ajog.2015.05.013. Epub 2015 May 14. | RAYYAN-INCLUSION: {"Adam"=>"Included", "David"=>"Included"}

10.1016/j.ajog.2015.05.013.

29.	Jairajpuri DS, Malalla ZH, Mahmood N, et al. Circulating microRNA expression as predictor of preeclampsia and its severity. *Gene* 2017; 627: 543-548. DOI: 10.1016/j.gene.2017.07.010. Epub 2017 Jul 8. | RAYYAN-INCLUSION: {"Adam"=>"Included", "David"=>"Included"}

10.1016/j.gene.2017.07.010.

30.	Biró O, Alasztics B, Molvarec A, et al. Various levels of circulating exosomal total-miRNA and miR-210 hypoxamiR in different forms of pregnancy hypertension. *Pregnancy Hypertension* 2017; 10: 207-212. DOI: 10.1016/j.preghy.2017.09.002.

31.	Youssef HMG and Marei ES. Association of MicroRNA-210 and MicroRNA-155 with severity of preeclampsia. *Pregnancy Hypertension* 2019; 17: 49-53. DOI: 10.1016/j.preghy.2019.05.010.

32.	Jelena M, Sopić M, Joksić I, et al. Placenta-specific plasma miR518b is a potential biomarker for preeclampsia. *Clin Biochem* 2020; 79: 28-33. DOI: 10.1016/j.clinbiochem.2020.02.012. Epub 2020 Feb 21. | RAYYAN-INCLUSION: {"Adam"=>"Included", "David"=>"Included"}

10.1016/j.clinbiochem.2020.02.012.

33.	Jairajpuri DS, Malalla ZH, Sarray S, et al. Analysis of differential expression of hypoxia-inducible microRNA-210 gene targets in mild and severe preeclamptic patients. *Non-coding RNA Research* 2021; 6: 51-57. DOI: 10.1016/j.ncrna.2021.03.001.

34.	Kolkova Z, Holubekova V, Grendar M, et al. Association of Circulating miRNA Expression with Preeclampsia, Its Onset, and Severity. *Diagnostics (Basel)* 2021; 11. DOI: 10.3390/diagnostics11030476. | RAYYAN-INCLUSION: {"Adam"=>"Included", "David"=>"Included"}

10.3390/diagnostics11030476.
